# Identification of Gene Co-Expression Networks Associated with Consensus Molecular Subtype-1 of Colorectal Cancer

**DOI:** 10.3390/cancers13225824

**Published:** 2021-11-20

**Authors:** Sha’Kayla K. Nunez, Corey D. Young, Ti’ara L. Griffen, Adaugo Q. Ohandjo, Lawrence P. McKinney, Scott Kopetz, James W. Lillard

**Affiliations:** 1 Department of Microbiology, Biochemistry & Immunology, Morehouse School of Medicine, Atlanta, GA 30310, USA; snunez@msm.edu (S.K.N.); COYoung@msm.edu (C.D.Y.); tiara.l.griffen@gmail.com (T.L.G.); LMckinney@msm.edu (L.P.M.); 2 East West Collaborative Research, Marietta, GA 30060, USA; adanmaqueen@gmail.com; 3 Department of Gastrointestinal Medical Oncology, The University of Texas M.D. Anderson Cancer Center, Houston, TX 77030, USA; skopetz@mdanderson.org

**Keywords:** colorectal cancer (CRC), consensus molecular subtype (CMS), microsatellite stability (MSI), weighted gene co-expression network analysis (WGCNA), mitogen-activated protein kinase (MAPK), Integromics

## Abstract

**Simple Summary:**

Colorectal cancer (CRC) is a frequently lethal disease with heterogenous outcomes. Alterations in the Wnt signaling pathways have been shown to promote activation of signaling pathways such as MAPK and PI3K-Akt. Consensus molecular subtyping (CMS) provides a cohesive structure to classify the heterogeneity of CRC using gene expression analysis. CMS is categorized into four subtypes: CMS1, immune; CMS2, canonical; CMS3, metabolic; and CMS4, mesenchymal. Here, we identify co-expressed gene networks associated with CMS1. Our findings distinguish co-expressed gene networks that play a pivotal role in key features specific for CMS1, such as immune infiltration and activation. The co-expressed gene networks for CMS1 were significantly and positively correlated with the TNF, WNT, and ERK1 and ERK2 signaling pathways. This study highlights the relevance of CMS1 gene networks relating to oncogenic signaling cascades, cell activation, and positive regulation of immune responses, promoting CRC progressiveness.

**Abstract:**

Colorectal cancer (CRC) is driven in part by dysregulated Wnt, Ras-Raf-MAPK, TGF-β, and PI3K-Akt signaling. The progression of CRC is also promoted by molecular alterations and heterogeneous—yet interconnected—gene mutations, chromosomal instability, transcriptomic subtypes, and immune signatures. Genomic alterations of CRC progression lead to changes in RNA expression, which support CRC metastasis. An RNA-based classification system used for CRC, known as consensus molecular subtyping (CMS), has four classes. CMS1 has the lowest survival after relapse of the four CRC CMS phenotypes. Here, we identify gene signatures and associated coding mRNAs that are co-expressed during CMS1 CRC progression. Using RNA-seq data from CRC primary tumor samples, acquired from The Cancer Genome Atlas (TCGA), we identified co-expression gene networks significantly correlated with CMS1 CRC progression. CXCL13, CXCR5, IL10, PIK3R5, PIK3AP1, CCL19, and other co-expressed genes were identified to be positively correlated with CMS1. The co-expressed eigengene networks for CMS1 were significantly and positively correlated with the TNF, WNT, and ERK1 and ERK2 signaling pathways, which together promote cell proliferation and survival. This network was also aligned with biological characteristics of CMS1 CRC, being positively correlated to right-sided tumors, microsatellite instability, chemokine-mediated signaling pathways, and immune responses. CMS1 also differentially expressed genes involved in PI3K-Akt signaling. Our findings reveal CRC gene networks related to oncogenic signaling cascades, cell activation, and positive regulation of immune responses distinguishing CMS1 from other CRC subtypes.

## 1. Introduction

Colorectal cancer (CRC) is characterized by uncontrolled cell growth in the colon or the rectum. CRC is the second most common cancer in the United States. The progression of CRC is promoted by molecular alterations and interconnectivity among gene mutations, chromosomal instability, transcriptomic subtypes, and immune signatures [1]. For the purpose of this manuscript, we define CRC progression as the activation of molecular pathways that regulate several biological processes such as cell proliferation and survival. Chromosomal instability in CRC represents about 80–85% of all cases, and it includes changes in essential genes such as APC, KRAS, and PI3K [2]. Deregulation of these pathways leads to an upregulation of proliferative and survival signals from the abnormal tumor microenvironment [3]. Mutations in APC, a key negative regulator of canonical Wnt signaling, lead to uncontrolled cell proliferation [4]. KRAS and PI3K mutations also promote uncontrolled cell proliferation through constitutive activation of mitogen-activated protein kinase (MAPK) signaling. MAPK signaling contributes to CRC progression by multiple means: differentiation regulation, cell proliferation, and gene transcription activation [5]. The MAPK pathway is reported to be vital to treating advanced cancers, as new MAPK inhibitors are improving survival for CRC [6]. First-line treatments of CRC were used in combination with second-line therapies such as cetuximab and panitumumab, which target EGFR [7].

There have been recent advances in novel therapies for CRC. Researchers are working on ways to better detect, classify, and predict outcomes for CRC. CRC is a heterogeneous disease; thus, characterizing molecular phenotypes is crucial for improving treatment efficacy and prognosis. Many approaches have been developed to create a classification for CRC based on gene expression heterogeneity; however, these were not successful in their clinical application. Studies have shown that conventional mutation-centered classification strategies do not fully explain the diversity in patient outcomes. For CRC, a more systematic, gene-based classification stratifies CRC patients better [8]. Since 2012, several research teams have employed bioinformatics methods for molecular subtyping; one of the largest international collaborative communities on CRC research is called the CRC Subtyping Consortium (CRCSC) [8]. Since their establishment in 2014, their work has led to a current consensus of a CRC classification system, consisting of six CRC subtyping systems derived from a single-omic research strategy [8]. This system identified four gene expression consensus molecular subtypes (CMS)—CMS1, CMS2, CMS3, and CMS4—using RNA-seq and multiple microarray datasets that included primary tumor samples from CRC patients [9]. Recently, an international effort of large-scale data sharing and analytics coordination compared six independent transcriptome-based CRC subtyping systems, which created four consensus molecular subtypes (CMS1-4) [1,9]. There are several challenges and emerging opportunities in using the CRC subtyping system in the clinical management of patients [10]. CRC subtypes CMS2 (37%) and CMS4 (23%) are more prevalent compared to CMS1 (14%) and CMS3 (13%). The most common subtypes are characterized by increased WNT and MYC signaling activation (CMS2) and TGFβ activation and angiogenesis (CMS4), whereas the least common are characterized by immune infiltration, hypermethylation, and microsatellite instability (CMS1) and epithelial and metabolic dysregulation (CMS2) [9]. Patients with microsatellite instability (MSI) tumors (mostly CMS1 tumors) do not benefit from single-agent treatments such as fluorouracil, but rather combination adjuvant therapy with fluorouracil, leucovarin, and oxaliplatin (FOLFOX) at stage III [11]. Further, stage III tumors at the time of diagnosis respond better to standard adjuvant therapy for CMS2 tumors [11], whereas CMS3 tumors do not benefit from therapeutic gene targets, but rather metabolic phenotypes, and CMS4 tumors are reported to not benefit from systemic adjuvant treatments and are resistant to anti-EGFR therapy when not associated with KRAS mutations [11]. In addition, the five-year overall survival rates for all stages differ amongst CMS types, with reported percentages of 77% for CMS2 and 73%, 75%, and 62% for CMS1, 3, and 4, respectively [11]. CMS2, having the highest five-year survival rate, is more commonly associated with left-sided tumors, unlike CMS1 tumors that are frequently associated with right-sided tumors and exhibit poor survival after relapse [11].

CMS1 patients have a worse prognosis due to having increased microsatellite instability and BRAF mutations [9]. Microsatellites are short tandem repeats that occur throughout both the coding and non-coding regions of the genome. These repetitive structures give rise to replication errors caused by DNA polymerase during replication. Four major repair proteins (MLH1, MSH2, MSH6, and PMS2) excise microsatellites. If any of these proteins lose function, loss of fidelity will occur. This loss in fidelity will lead to errors in the genome not being corrected, which accumulate to cause a deficiency in the mismatch repair system [12,13]. MSI, or deficiency of the DNA mismatch repair system (dMMR), occurs in about 15% of CRCs. CMS1 tumors are characterized by the overexpression of DNA damage repair, microsatellite instability, and immune response proteins and is associated with higher prevalence of BRAF mutations, young and female patients, and right-sided tumors [9,14]. This manuscript aims to improve understanding of the molecular and genomic features associated with CMS1. Herein, we characterize the gene expression signatures and associated co-expressed gene networks during CMS1 CRC progression. Using RNA sequencing data from CRC primary tumor samples, acquired from The Cancer Genome Atlas (TCGA), we identified co-expression gene networks using weighted gene co-expression network analysis (WGCNA) correlated with CMS1 CRC progression.

## 2. Materials and Methods

### 2.1. Data Collection and Gene Network Analysis

TCGA CRC patient RNA-seq data were acquired from GDC via the TCGA data portal (https://portal.gdc.cancer.gov, accessed on 4 November 2021). Download date for network analysis was on or before 5 October 2018. FPKM (fragments per kilobase of exon per million mapped fragments) data were downloaded for each participant along with clinical data. Network analysis was completed via the data mining method WGCNA (Weighted Gene co-expression Network Analysis) *r* package (version v1.70.3). Methods for data normalization, removal of outliers, batch effect correction, co-expression network analysis, differential expression analysis, and gene ontology (GO) enrichment analysis were adapted from “Transcriptome Network Analysis Identifies CXCL13-CXCR5 Signaling Modules in the Prostate Tumor Immune Microenvironment”, accessible at https://doi.org/10.1038/s41598-019-46491-3 (accessed on 4 November 2021) [15,16,17].

### 2.2. Pathway Analysis

Pathview is an online package toolset, accessible at pathview.uncc.edu, for pathway-based data integration and visualization. This tool maps and renders imported data on relevant pathway graphs that are automatically downloaded and mapped to a pathway. This tool integrates pathways and gene set enrichment analysis [18,19].

### 2.3. Gene Expression Analysis

Genome-Scale Integrated Analysis of Networks in Tissues (GIANT) is an online tool, accessible at hb.flatironinstitute.org, or predictions of tissue-specific gene expressions and detections based on a gene list. Genes within a cluster share a local network neighborhood and form a specific functional module [20].

## 3. Results

**Figure 1:**
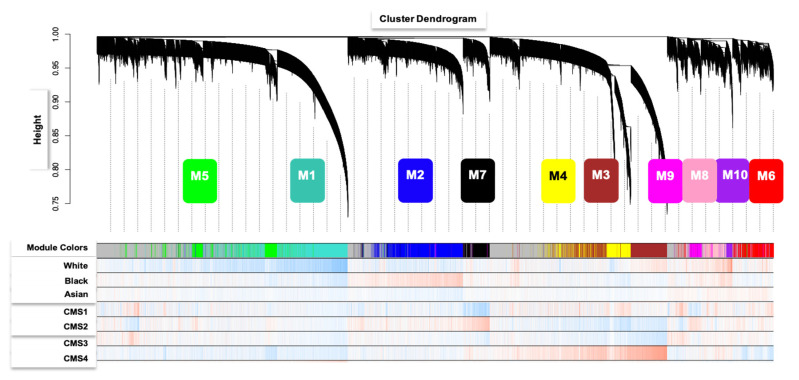
Gene dendrogram of clustered dissimilarity and module colors, based on consensus topological overlap. The gene dendrogram was obtained by average linkage hierarchical clustering. The module colors underneath the dendrogram show 10 module assignments determined by the Dynamic Tree Cut, which contains a group of highly connected genes. Clinical trait relationships were assessed for each color-coded module. Bypassing the default Pearson correlation method in WGCNA, we applied biweight mid-correlation as a robust alternative implemented in the WGCNA function (bicor). Module color bicor color scale (−1, blue; 0, white; +1, red) represents modules with significant student’s p cluster.

Four hundred and seventy-eight primary CRC tumor transcriptomes were acquired from The Genomic Data Commons (GDC). The CRC patient-specific RNA-seq data obtained from GDC are a Cancer Genome Atlas (TCGA) dataset. The gene expression data, from each patient, were used to determine the co-expressed gene networks that are positively correlated with CMS1 versus non-CMS1 subtypes of CRC, and how they correlate with clinical and biological traits of CRC cases, and patient demographics. Weighted Gene Co-expression Network Analysis (WGCNA) was utilized to define gene co-expression networks and then these were correlated with clinical outcomes such as CMS, microsatellite instability (MSI), and tumor region. This established clustering and dimensionality reduction method defined 10 modules, which were assessed for association with qualitative patient characteristics including gender and race. Genes with statistically significant correlations to relevant traits. Particular attention was paid to determining the role of co-expressed gene networks positively correlated with CMS1 genes in CRC. First, we characterized gene co-expression networks in CRC. Overall, 10 gene network modules were formed from applying WGCNA to the 456 CRC primary tumor samples (Figure 1). We have reported stages for all 456 patients as a supplementary table in Table S2. Of the 456 patients, 18 patients had stage 0 CRC, defined as in situ; 72 patients had stage 1; stage 174 patients had stage 2; 128 patients had stage 3; and 64 patients had stage 4 CRC. A total of 78% of TCGA CMS1 patients had stage 2–4 CRC.

**Figure 2:**
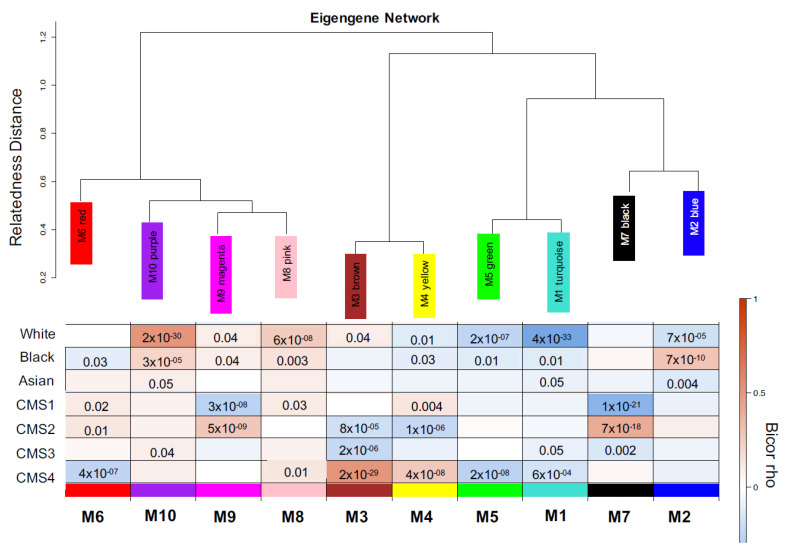
(Top) Hierarchical clustering of module eigengenes that represent modules found after CRC TCGA clustering analysis. Eigengenes positively correlated are grouped together by branches of the dendrogram. (Bottom) Heatmap plot of the adjacencies in the eigengene network including the trait weight. Each row and column in the heatmap correspond to one module eigengene (labeled by color) or weight. In the heatmap, blue color represents low adjacency (negative correlation), while red represents high adjacency (positive correlation).

To identify clinical associations, module expression was correlated (bicor-rho) with quantitative and qualitative clinical traits. Co-expression modules M6 (red), M8 (pink), and M4 (yellow) were significantly and positively associated with CMS1, as depicted in Figure 2.

**Figure 3:**
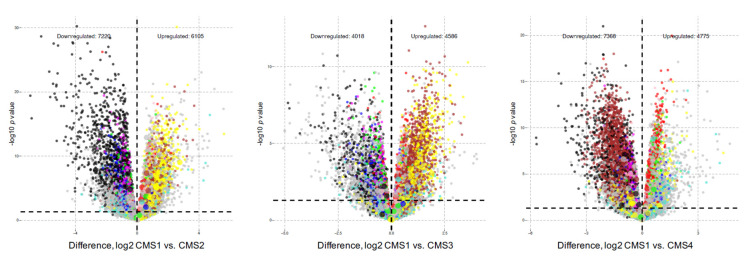
Volcano plots of differential gene expression identifies gene transcripts that contain upregulated genes and downregulated genes based on CMS status for each module. The number of downregulated gene transcripts is denoted on the left and number of upregulated gene transcripts is denoted on the right. The X axis represents the Log_2_ fold-change and the Y-axis represents the negative Log_10_ *p*-value. Gene transcripts are colored by module membership (light grey dots, no module assignment).

WGCNA modules M6 (red), M8 (pink), and M4 (yellow) were significantly and positively associated with CMS1. Genes within modules M6, M8, and M4 are differentially expressed and upregulated in CMS1 compared to other subtypes (Figure 3).

The co-expressed genes within modules M6, M8, and M4 were positively associated with lymphocyte activation, T-cell receptor signaling, TNF signaling pathways, and positive regulation of cytokines (Table 1).

The genes identified in the CMS1 TCGA positively correlated modules included CCL19, CXCL13, FCRN1, IFNG, IL-17A, TNFRSF17, and XCL1. These genes are associated with immunoregulatory interactions between lymphoid and non-lymphoid cells, B cell receptor signaling, and tertiary lymphoid structures (TLS). The development of conventional lymph nodes (LN) depends on interaction between CD4− CD3− lymphoid tissue inducer (LTi) cells. This leads to expression of CCL19 and CXCL13, which as a localized concentration gradient attracts additional LTi cells, and the recruitment and positioning of T and B cells. CCL19 plays an important role in the trafficking of T cells in the thymus, and in T cell and B cell migration to secondary lymphoid organs. 

Pivotal studies have shown that a lymphocytic reaction to CRC is associated with MSI [21], a significant characteristic of CMS1. CMS1 is enriched for MSI and is defined by the overexpression of genes associated with cytotoxic lymphocytes [21]. Infiltrating lymphocyte markers such as CD80, CD8, CD4, and CD68 were found in the CMS1 associated modules. Infiltrating lymphocyte markers, found within CMS1 positively correlated modules, were co-expressed with genes associated with high T and B cell infiltration. Indeed, the highly expressed genes included lymphocyte-attracting chemokines, e.g., CXCR6, CXCL9, CXCL10, CX3CL1 (for T cells), and CXCL13 (for B cells). We identified a plethora of chemokine receptors in CMS1 TCGA positively correlated modules (CCR1, CCR2, CCR3, CCR5, CCR8, CXCR1, CXCR2, and CXCR4), each of which plays a significant role in recruiting leukocytes to inflammatory sites [22].

Inflammation in CRC is thought to involve cross-talk between immune cells, pro-inflammatory mediators, chemokines, and cytokines that leads to the activation of certain pathways such as NF-κβ and the JAK-STAT pathway leading to tumor cell proliferation and growth [23]. IL6, IL10, IL1β, and TNFRSF10A were positively correlated with CMS1 and are associated with T helper cell activation through the NF- κβ pathway [24]. Most CRC tumors have constitutive activation of transcription factors that activate inflammatory pathways, such as NF-κβ and the JAK-STAT pathway [25]. The JAK-STAT pathway is a major transducer of cytokine-signaling regulating inflammation and immune responses in CRC [26]. The JAK-STAT pathway is made of eight STAT proteins; primarily STAT-1 and -2 are involved in immune responses [26]. JAK-STAT activation is a notable characteristic of CMS1 as it displays strong immune activation [9]. MAPK signaling has also been reported to be induced by hypermutated CMS1 [9,27]. Constitutive activation of MAPK leads to CRC progression by regulating CRC cellular proliferation and differentiation, causing chemotherapy resistance, and activating STAT-1 [27,28]. MAPK is one of the most prominent pathways for cellular proliferation and communicates with other pathways such as PI3K-Akt [29].

**Figure 4:**
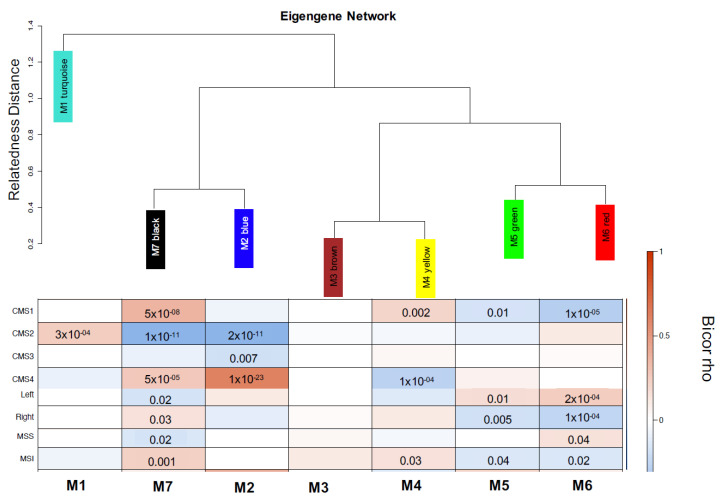
(Top) Hierarchical clustering (top) of module eigengenes that represent the modules found in the clustering analysis for Integromics. Eigengenes that were positively correlated are grouped together by branches of the dendrogram. (Bottom) Heatmap plot of the adjacencies in the eigengene network including the trait weight. Each row and column in the heatmap correspond to one module eigengene (labeled by color) or weight. In the heatmap, blue color represents low adjacency (negative correlation), while red represents high adjacency (positive correlation).

To independently validate this CMS1-elevated cytokine gene signature found in the TCGA dataset, we used a microarray dataset of 278 individual tumor sample transcriptomes, called Integromics, acquired from the MD Anderson Cancer Center. Stages for our Integromics cohort is provided as supplementary material in Table S2. All Integromics CMS1 patients had stage 3 or 4 CRC. WGCNA CMS1 Integromics modules M7 and M4 were positively correlated with CMS1 status of distinct individuals’ tumors (Figure 4). These modules were also positively correlated with right-sided tumors and MSI. It was previously reported that right-sided tumors are characterized by high MSI, and BRAF mutations [30,31], key features of CMS1.

Right-sided tumors in CRC have been significantly associated with poorer survival of patients. Previous reports validated a strong association between BRAF mutant CRC and MSI, having poorer survival and a greater propensity for metastatic spread [32]. BRAF was found in our TA WGCNA M8 module, which was also positively associated with immune-driven CMS1, as well as CMS4, which represents higher chromosomal instability, and within African-American race. Of our three CMS1-positive TCGA modules, only M8 showed a strong correlation to African-American race. Evidence has shown that African-Americans tend to present more right-sided tumors compared to non-Hispanic whites [33], but this tendency arises through a chromosomal instability-associated molecular pathway rather than through MSI or hypermethylation. A strong correlation to CMS4, with inherent chromosomal instability, could in part explain why the immune-driven TCGA CMS1- associated M8, but not M6 or M4 is associated with African-American race. CMS1 and CMS4 tumor subgroups have high expression of lymphoid and myeloid signatures, in addition to displaying a strong immune and strong inflammatory profile compared to the relative expression of this module in CMS2 and CMS3 tumor subgroups, as previously reported [34]. 

**Figure 5:**
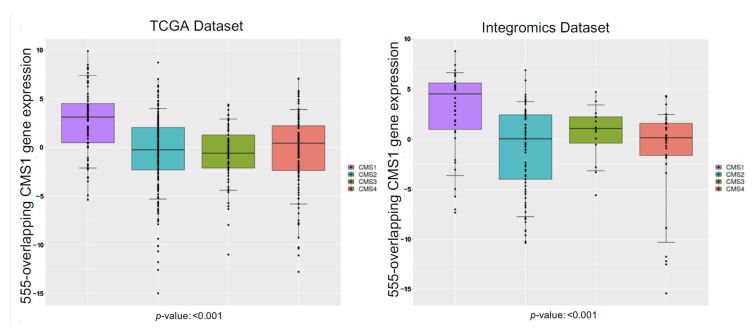
Box-plot distribution analysis of the 555-overlapping CMS1 gene signature, common to TCGA and Integromics datasets. This gene signature was compared across the four consensus molecular subtypes (CMS1, CMS2, CMS3, and CMS4). The y-axis measures the z score relative to the gene expression, horizontal lines define minimum and maximum values, and dots define outliers. The 555- overlapping genes are significantly distributed in CMS1 for both the TCGA and Integromics datasets. One-way ANOVA analysis was used for the statistics to generate *p*-values and any potential outliers.

**Figure 6:**
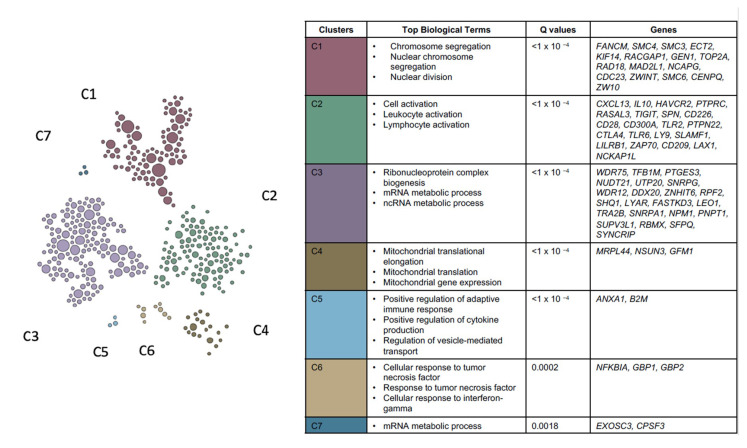
Genome scale integrated analysis (GIANT) used to detect cohesive gene clusters from the 555-overlapping CMS1 gene signature list. Genes within a cluster share local network neighborhoods. From the 555 overlapping genes, seven functional clusters (C1–7) were detected from the top significant genes with their reported top biological processes.

**Figure 7:**
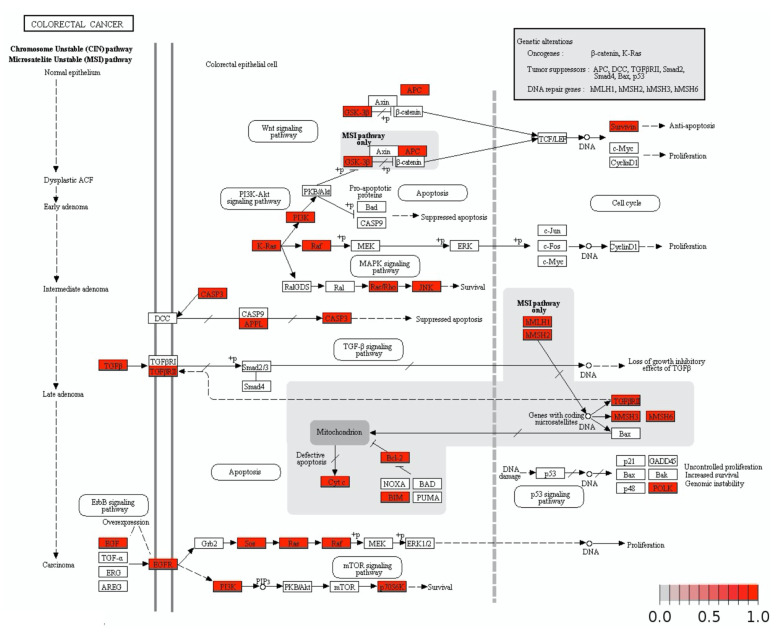
Integrated pathway analysis of the 555-overlapping CMS1 gene signature showing positive correlations with numerous genes in CRC signaling pathways. The 555 overlapping genes have strong positive correlations with gene regulators that activate PI3K-AKT signaling, ErbB signaling, TGFβ signaling pathways, and mismatch repair genes.

**Figure 8:**
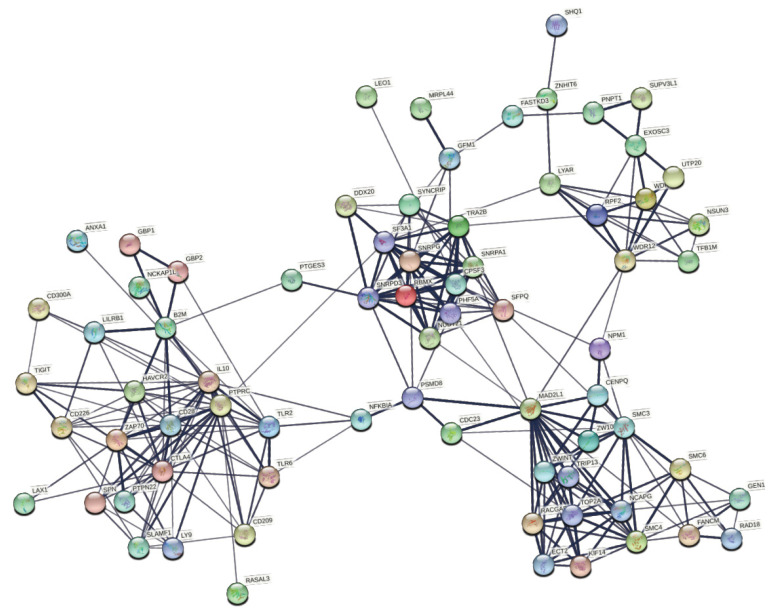
Predicted protein–protein interaction of immune regulators assigned to the 555 overlapping CMS1 gene signature. Colored nodes represent query proteins and first shell interactors. Lines between each node represent protein–protein associations and contribute to a shared function between proteins. Line thickness signifies confidence, where thicker lines equate to stronger confidence in the protein–protein association.

The TCGA M4, M6, and M8 modules included 555 genes overlapping with members of the Integromics M4 and M7 modules, associated with CMS1 (Figure 5). These 555 genes were considered as a loosely defined CMS1 gene signature, we report these genes in our supplementary material in Table S1. Genome-Scale Integrated Analysis of Networks in Tissues (GIANT) analysis was used to group these genes into smaller clusters to better understand their function and potential interactions (Figure 6). This CMS1 gene signature was shown to be associated with leukocyte activation, positive regulation of adaptive immune response, and positive regulation of cytokine production. We used Pathview to identify the CRC pathways associated with the 555 overlapping CMS1 gene signature (Figure 7). The signature list of genes is enriched with members of the MAPK, TGFβ, MSI, and PI3K-AKT pathways. Interestingly, the PI3K-AKT pathway has not been associated with the CMS1 phenotype until now. Several mutations in CRC have been reported to occur upstream of EGFR and downstream of MAPK [29]. Several key gene products of the MAPK pathway were found to be associated with several immune-response-activating genes from the 555 overlapping CMS1 gene signature (Figure 8).

Mitogen-activated protein kinases (MAPKs) are a family of protein Ser/Thr protein kinases that convert extracellular stimuli into a wide range of cellular responses. MAPKs function to regulate survival, cell proliferation, motility, and differentiation. The MAPK pathway is critical in CRC, typically activated by cytokines and chemokines. Once this pathway is activated, the MAPK involved phosphorylates different substrates in the cytosol and nucleus to execute a biological response. This biological response often involves cell survival. Cascade of ERK1 or ERK2 (MAPK2 or MAPK1, respectively), each phosphorylation-dependently activated downstream in the MAPK pathway, is upstream of the phosphorylation of different cytoskeletal proteins that affect cell movement and cell adhesion [35].

## 4. Discussion

The transmembrane protein EGFR belongs to the ErbB family of receptors of tyrosine kinases and is one of the most significant upstream receptors to activate the MAPK pathway. The 555-overlapping CMS1 gene signature contains a plethora of G protein-coupled receptors (GPCRs), cytokines and integrins, listed in Figure 7, all of which are critical for EGFR activation. In turn, these factors promote transcription and CRC cell proliferation [5,28]. MAPK positive regulators TRIBS, DUSP1, MAP3K8, CRK, PTPN7, CMKLR1, CNDP2, MAPK11, GPR31, FCRL3, and CARD9 were found in the 555-overlapping CMS1 gene signature. Stimulation of these MAPK regulators within our CMS1 signature leads to the activation of the JNK (MAPK8 or MAPK9) and ERK1/ERK2 pathways. These regulators promote B cell proliferation, and cell adhesion, and are they critical for producing TNF-α during immune responses. These MAPK regulators are also co-expressed with pro-inflammatory cytokines such as IL-1β (TCGA M4) that are known for causing resistance to EGFR-targeted therapies [36]. Activation of this ErbB receptor leads to auto-phosphorylation and release of the Grb2/SOS complex, which in turn activates the RAS and PI3K pathways [34]. Within this context, we have shown that MAPK regulators are positively correlated with the CMS1 phenotype.

PI3K regulators PIK3C3, PPP1R16B, CMKLR1, PIK3AP1, PIK3CG, and PIK3CD were also positively correlated with CMS1 in our dataset. These PI3K regulators activate signaling cascades involved in cell growth, morphology, motility, survival, and proliferation. The PI3K-Akt pathway is a critical pathway in CRC as it has been reported that phosphorylation of Akt in CRC correlates with cell death inhibition and cell proliferation [37]. In addition to PI3K regulators causing activation of this pathway, interleukins and chemokines play a major role as well. Within the 555-overlapping CMS1 gene signature, we found IL6 and IL6R, which have been previously reported to activate PI3K-Akt. Transient phosphorylation of STAT3^Tyr705^ is caused by IL-6 signaling, which leads to phosphorylation of Akt^Ser473^ and ERK1/2^Thr202/tyr204^ [38]. IL-6 also leads to the release of the pro-inflammatory chemokine CCL2 [38] which is meditated via JAK-STAT3, a pathway attributable to CMS1, and PI3K-Akt, a pathway atypical of CMS1 subgroup assignment. Several chemokines have been shown to activate MAPK and PI3K signaling pathways leading to cell growth, migration, and transcription activation [39]. Signaling via the CMS1-associated CXCL13, and its receptor CXCR5, has been shown to induce cancer progression through PI3K, Akt, ERK 1/2, and Jun [40,41]. Indeed, others have shown that CXCL13 stimulates PI3K-Akt activation and increases the secretion of MMP-13 in CRC [42]. Upon activation of chemokine receptor-mediated signaling, the PI3K molecules serve as central signaling molecules: chemokine receptors are coupled to heterothermic G proteins α,β,γ, activating Class1A and 1B PI3Ks [39,41]. Our analysis shows a 555-overlapping CMS1 gene signature to be involved in cell signaling associated with CRC progression through PI3K, Akt, ERK 1/2, and Jun.

CMS1 having genes involved in PI3K-AkT signaling is a process atypically assigned to CMS1 CRCs. Our findings highlight the relevance of gene networks involved in CMS1 as it relates to oncogenic signaling cascades, cell activation and positive regulation of immune responses associated with CMS1 CRC progression.

## 5. Conclusions

The CMS1 phenotype accounts for 14% of CRC tumors, is characterized by a high immune response, and is associated with right-sided tumors and MSI. Herein we aimed to better understand the factors that contribute to the CMS1 phenotype. Genomic studies have shown that EGFR and downstream MAPK and PI3K signaling pathways are nearly ubiquitous events in CRC. Our findings strongly support CMS1 positively correlated modules, and its co-expression network activates important pathways such as JAK-STAT, MAPK, and PI3K-Akt, to be associated with CRC progression and tumor growth. These pathways have frequently been associated with CRC progression due to their ability to activate signaling cascades of Ras-Raf-ERK signaling that contribute to cell proliferation, differentiation, and survival. Our findings highlight the relevance of gene networks involved in CMS1 as it relates to oncogenic signaling cascades, cell activation, and positive regulation of immune responses associated with CMS1 CRC progression. For the first time, we identified a strong correlation of 555-overlapping genes for CMS1 gene signature involved in chemokine-mediated signaling pathways, immune responses, inflammatory responses, and cell activation. Despite extensive studies on signaling pathways associated with CRC progression, we identified 555-overlapping genes found in critical molecular pathways known to progress colorectal cancer which are involved in immune hypermutated CMS1. Understanding how co-expressed genes and their networks are associated with CRC clinical phenotypes and biological functions will yield new insights into understanding biomarkers and more targeted therapies.

## Figures and Tables

**Table 1:** Biological processes of module associations for CMS1. Gene Ontology (GO) elite analysis was used to determine the top 5 biological processes of each module positively associated with CMS1 compared to modules that were negatively associated with CMS1.

	Module	Top 5 Biological Processes	False Discovery Rate
**Positively correlated with CMS1**	Yellow *p*-value = 0.004	Immune system process Immune response Regulation of immune system process Lymphocyte activation Regulation of immune response	5.19 × 10^−170^ 3.83 × 10^−163^ 1.19 × 10^−121^ 9.75 × 10^−115^ 2.21 × 10^−101^
	Red *p*-value = 0.02	Cellular nitrogen compound metabolic process Cellular metabolic process Cell cycle Metabolic process Cell cycle process	2.61 × 10^−34^ 2.61 × 10^−34^ 1.25 × 10^−31^ 5.87 × 10^−30^ 3.40 × 10^−26^
	Pink *p*-value = 0.03	Cellular macromolecule metabolic process Macromolecule metabolic process Regulation of nucleobase-containing compound metabolic process T cell receptor signaling Regulation of cellular macromolecule biosynthetic process	9.43 × 10^−38^ 1.38 × 10^−34^ 5.40 × 10^−30^ 5.40 × 10^−30^ 2.69 × 10^−29^
**Negatively correlated with CMS1**	Black *p*-value = 1 × 10^−21^	Transcription, DNA-templated Metabolic process Biosynthetic process Heterocycle biosynthetic process Aromatic compound biosynthetic process	0.0084 0.0084 0.0084 0.0084 0.0084
	Magenta *p*-value = 3 × 10^−8^	Nucleic acid metabolic process RNA metabolic process Cellular nitrogen compound metabolic process Nucleobase-containing compound metabolic process Macromolecule metabolic process	1.96 × 10−12 1.77 × 10−10 1.77 × 10−10 2.69 × 10−10 2.73 × 10−10

## Data Availability

The TCGA COAD datasets downloaded during and/or analyzed during the current study are available from the corresponding author on reasonable request.

## Supplementary Materials

The following are available online at https://www.mdpi.com/article/10.3390/cancers13225824/s1, Table S1: 555-overlapping genes for CMS1 gene signature, Table S2: Patient stages.
