# Supplementary material for: Identification of Gene Co-Expression Networks Associated with Consensus Molecular Subtype-1 of Colorectal Cancer
Source: Cancers (Basel). 2021 Nov 20;13(22):5824. doi: 10.3390/cancers13225824 (PMC8616344; doi:10.3390/cancers13225824)
Supplement: Supplementary file 1 [file cancers-13-05824-s001.zip › Table S1- 555 overlapping CMS1 Genes .pdf]

555-overlapping genes for CMS1 gene signature	
**1.**	IKZF1
**2.**	IL10RA
**3.**	CD53
**4.**	ARHGAP9
**5.**	NCKAP1L
**6.**	KLHL6
**7.**	SLA
**8.**	BTK
**9.**	PTPRC
**10.**	RHOH
**11.**	STX11
**12.**	RCSD1
**13.**	RASAL3
**14.**	TAGAP
**15.**	LY9
**16.**	LILRB1
**17.**	TNFAIP8L2
**18.**	TRAF3IP3
**19.**	GIMAP6
**20.**	TBC1D10C
**21.**	GMFG
**22.**	SAMSN1
**23.**	ARHGAP15
**24.**	HAVCR2
**25.**	ITK
**26.**	DOCK10
**27.**	CD300A
**28.**	ARHGEF6
**29.**	PLEK
**30.**	CD247
**31.**	CLEC7A
**32.**	DPEP2
**33.**	CD28
**34.**	IRF4
**35.**	LAX1
**36.**	TIGIT
**37.**	EVI2A
**38.**	MS4A4A
**39.**	ZNF831
**40.**	CHST2
**41.**	CR1
**42.**	CD3G
**43.**	CASS4

**44.**	DOK3
**45.**	SIRPB2
**46.**	TFEC
**47.**	GIMAP8
**48.**	GIMAP7
**49.**	UBASH3A
**50.**	SIGLEC1
**51.**	CD226
**52.**	PYHIN1
**53.**	NLRC3
**54.**	KCNK13
**55.**	WDFY4
**56.**	ITGAX
**57.**	SPN
**58.**	CHST11
**59.**	CTLA4
**60.**	CD80
**61.**	LILRB5
**62.**	STAT4
**63.**	CD274
**64.**	TLR6
**65.**	IL7R
**66.**	IL18RAP
**67.**	IL10
**68.**	DOCK8
**69.**	SLC1A3
**70.**	SYTL3
**71.**	C5AR1
**72.**	NLRP3
**73.**	SLFN11
**74.**	GBP1
**75.**	LRRC8C
**76.**	CLECL1
**77.**	RASGRP3
**78.**	ANKRD44
**79.**	KLRG1
**80.**	TLR2
**81.**	STAT2
**82.**	CLEC4D
**83.**	BATF3
**84.**	RGS1
**85.**	PTPN22
**86.**	LRRK2
**87.**	SH2B3
**88.**	KYNU

**89.**	PDE4B
**90.**	HMOX1
**91.**	PIM2
**92.**	PLCB2
**93.**	CD36
**94.**	FLVCR2
**95.**	CYTH4
**96.**	SLAMF1
**97.**	GAB3
**98.**	LILRB2
**99.**	RASSF5
**100.**	SLC9A9
**101.**	CXorf21
**102.**	ICOS
**103.**	CD209
**104.**	DPYD
**105.**	EVL
**106.**	CLEC4A
**107.**	MEI1
**108.**	CD69
**109.**	APOBEC3G
**110.**	FCRL5
**111.**	ENPP2
**112.**	PLCG2
**113.**	ITGA4
**114.**	CLIC2
**115.**	FCGR2A
**116.**	RASGRP2
**117.**	BIN2
**118.**	ACP5
**119.**	APOBEC3H
**120.**	ATP8B4
**121.**	TRIM22
**122.**	GNG2
**123.**	ZAP70
**124.**	PREX1
**125.**	FCRL2
**126.**	LILRA6
**127.**	HLA-DOB
**128.**	APOL3
**129.**	GLT1D1
**130.**	LPXN
**131.**	SAMD9L
**132.**	CSF2RA
**133.**	FCGR3B

**134.**	CLNK
**135.**	SLC31A2
**136.**	CLEC2D
**137.**	SAMHD1
**138.**	MIAT
**139.**	ADAM8
**140.**	RASSF4
**141.**	ZBTB32
**142.**	TSPAN32
**143.**	IL18R1
**144.**	MS4A14
**145.**	GBP2
**146.**	DRAM1
**147.**	CD83
**148.**	ARRDC5
**149.**	SOCS3
**150.**	TIAM1
**151.**	PARP9
**152.**	IFI16
**153.**	XAF1
**154.**	IRAK3
**155.**	LGALS12
**156.**	TRIM69
**157.**	BIRC3
**158.**	ST3GAL5
**159.**	C9orf139
**160.**	JAK2
**161.**	MIR155HG
**162.**	SCPEP1
**163.**	LHFPL2
**164.**	C22orf34
**165.**	B2M
**166.**	HHEX
**167.**	ADCY7
**168.**	ERMN
**169.**	ITGAE
**170.**	PATL2
**171.**	MFSD1
**172.**	CCL7
**173.**	MAP3K8
**174.**	NFKBIA
**175.**	BTN2A2
**176.**	CREM
**177.**	KLRK1
**178.**	KAT2B

**179.**	ANXA1
**180.**	CMTM2
**181.**	HRK
**182.**	FBXO15
**183.**	RILPL2
**184.**	TXK
**185.**	RGS10
**186.**	BMP2K
**187.**	PTGES3
**188.**	MAD2L1
**189.**	GLO1
**190.**	CCDC59
**191.**	MRPL42
**192.**	BRIX1
**193.**	ABCE1
**194.**	MRPL1
**195.**	HAT1
**196.**	RSL24D1
**197.**	AMD1
**198.**	RAD51AP1
**199.**	CENPK
**200.**	NUP37
**201.**	GLMN
**202.**	ANAPC10
**203.**	BCAS2
**204.**	VRK1
**205.**	PPP1CC
**206.**	PI4K2B
**207.**	CENPQ
**208.**	GRPEL2
**209.**	TMPO
**210.**	LRRC40
**211.**	GPN3
**212.**	CHAC2
**213.**	MRPS35
**214.**	CYCS
**215.**	AIMP1
**216.**	SMNDC1
**217.**	NUP35
**218.**	MRPL35
**219.**	MRPL50
**220.**	TFAM
**221.**	MRPL44
**222.**	MRPL39
**223.**	MND1

**224.**	CCT8
**225.**	ACTR6
**226.**	UBA3
**227.**	RPF1
**228.**	NCAPG
**229.**	PPP2CA
**230.**	SLBP
**231.**	VTA1
**232.**	NPM1
**233.**	METAP2
**234.**	MOCS2
**235.**	NAE1
**236.**	PSMA4
**237.**	ECT2
**238.**	NOC3L
**239.**	DCLRE1A
**240.**	TRA2B
**241.**	NUDT21
**242.**	MTHFD2L
**243.**	PRDX3
**244.**	TPRKB
**245.**	FASTKD3
**246.**	SNRPG
**247.**	ZCCHC9
**248.**	UBE2T
**249.**	DNA2
**250.**	RAD1
**251.**	ATG5
**252.**	PRKRA
**253.**	RAD54B
**254.**	LIN7C
**255.**	POLE2
**256.**	UTP18
**257.**	EIF3M
**258.**	API5
**259.**	WDR12
**260.**	CCT2
**261.**	SKP2
**262.**	ATG4C
**263.**	TFB2M
**264.**	PAICS
**265.**	DLAT
**266.**	ZC3H15
**267.**	ACP1
**268.**	PSMA3

**269.**	CCDC138
**270.**	BCCIP
**271.**	ERCC8
**272.**	WDR75
**273.**	NEIL3
**274.**	COQ3
**275.**	EEF1E1
**276.**	EXOSC9
**277.**	RPAP3
**278.**	UBE2D3
**279.**	RPF2
**280.**	PSMD14
**281.**	BTF3L4
**282.**	PPA2
**283.**	GFM2
**284.**	TBCA
**285.**	RFC4
**286.**	G2E3
**287.**	TIMM23
**288.**	PEX3
**289.**	HSPA14
**290.**	TRIAP1
**291.**	CDC23
**292.**	DCUN1D5
**293.**	UBA5
**294.**	DDX20
**295.**	RSRC1
**296.**	SASS6
**297.**	MELK
**298.**	IPO11
**299.**	PLRG1
**300.**	LYAR
**301.**	PSMA5
**302.**	TXNDC12
**303.**	PNPT1
**304.**	EXOC6
**305.**	FANCL
**306.**	PDHX
**307.**	CUL2
**308.**	SHQ1
**309.**	CCNH
**310.**	LLPH
**311.**	VDAC3
**312.**	POLR3G
**313.**	RBMX

**314.**	RACGAP1
**315.**	C17orf75
**316.**	ETF1
**317.**	PSMD6
**318.**	TIMM17A
**319.**	METTL5
**320.**	CDC123
**321.**	GTF2B
**322.**	BTG3
**323.**	PPP1R8
**324.**	PLK4
**325.**	MRPS30
**326.**	CDC7
**327.**	SLC25A40
**328.**	CASP3
**329.**	MASTL
**330.**	DNTTIP2
**331.**	SYNCRIP
**332.**	ZW10
**333.**	SVIP
**334.**	PDSS1
**335.**	GTF2H1
**336.**	RAD18
**337.**	RTKN2
**338.**	PCGF6
**339.**	NOL7
**340.**	TFB1M
**341.**	MGST1
**342.**	EBNA1BP2
**343.**	CCDC43
**344.**	NUP107
**345.**	CPSF3
**346.**	ZWINT
**347.**	C5orf34
**348.**	PDE12
**349.**	EIF3J
**350.**	TIAL1
**351.**	TRMT11
**352.**	DONSON
**353.**	MRPS9
**354.**	INTS12
**355.**	C12orf4
**356.**	HSPD1
**357.**	C3orf33
**358.**	SLC30A9

**359.**	NUDT5
**360.**	TRDMT1
**361.**	SUPV3L1
**362.**	PAFAH1B2
**363.**	BZW1
**364.**	COPS8
**365.**	SCLT1
**366.**	DDX21
**367.**	DEPDC4
**368.**	MRPS23
**369.**	HSPE1
**370.**	BRIP1
**371.**	CEPT1
**372.**	ATG12
**373.**	INTS7
**374.**	KIF15
**375.**	SLC35A3
**376.**	SNRNP40
**377.**	SNRPA1
**378.**	METTL2A
**379.**	CSNK1G3
**380.**	ILF2
**381.**	TOP2A
**382.**	TADA1
**383.**	PCMT1
**384.**	PRDX1
**385.**	GLRX2
**386.**	GART
**387.**	LRPPRC
**388.**	THUMPD3
**389.**	STIL
**390.**	SAAL1
**391.**	RPAP2
**392.**	FBXO45
**393.**	MCM6
**394.**	GFM1
**395.**	HELLS
**396.**	FANCM
**397.**	DHX15
**398.**	RNF14
**399.**	NECAP1
**400.**	MED7
**401.**	DNAJC25
**402.**	TMEM167A
**403.**	ZDHHC6

**404.**	MFN1
**405.**	WDHD1
**406.**	L2HGDH
**407.**	SLC33A1
**408.**	FBXO22
**409.**	HIBCH
**410.**	MNAT1
**411.**	TRIT1
**412.**	GSPT1
**413.**	YARS2
**414.**	CEP70
**415.**	ARL6
**416.**	CRYZL1
**417.**	BUB1B
**418.**	MTRF1L
**419.**	VRK2
**420.**	SAR1A
**421.**	PGM2
**422.**	CHUK
**423.**	ANLN
**424.**	VDAC2
**425.**	PGRMC2
**426.**	TAF5
**427.**	DNM1L
**428.**	FAM111B
**429.**	HSPA9
**430.**	GAR1
**431.**	ZZZ3
**432.**	TYW3
**433.**	NSMCE4A
**434.**	UMPS
**435.**	ATG3
**436.**	SLC10A7
**437.**	EXOSC3
**438.**	MTO1
**439.**	MRPL51
**440.**	ASCC1
**441.**	ZNF354A
**442.**	NR2C1
**443.**	NCBP1
**444.**	RFESD
**445.**	FBXO28
**446.**	MINPP1
**447.**	ZRANB3
**448.**	SNRNP48

**449.**	SENP8
**450.**	UEVLD
**451.**	RPP38
**452.**	ERI1
**453.**	CMAS
**454.**	LDHB
**455.**	NFXL1
**456.**	METTL8
**457.**	PPP2R1B
**458.**	TXNRD1
**459.**	RABGGTB
**460.**	AGFG1
**461.**	GPD2
**462.**	MCCC2
**463.**	LRRC8D
**464.**	RNF2
**465.**	POLR2B
**466.**	NUP160
**467.**	LDHA
**468.**	ATAD5
**469.**	NDUFS1
**470.**	MSH3
**471.**	IDE
**472.**	MAPK9
**473.**	ACADSB
**474.**	AMN1
**475.**	SMYD2
**476.**	CRYZ
**477.**	MSH6
**478.**	HSD17B4
**479.**	AASDH
**480.**	GTPBP4
**481.**	SFPQ
**482.**	HSPA4L
**483.**	ARV1
**484.**	BDP1
**485.**	RIF1
**486.**	USP8
**487.**	TRPM7
**488.**	PRPF38B
**489.**	PANK3
**490.**	CEP97
**491.**	MATR3
**492.**	SMC6
**493.**	SMC4

**494.**	MED1
**495.**	ZNF326
**496.**	ARHGAP21
**497.**	CHD1
**498.**	SMC3
**499.**	NHLRC2
**500.**	STAG1
**501.**	NEDD1
**502.**	LARP4
**503.**	STX17
**504.**	PPP4R2
**505.**	XRN1
**506.**	PGGT1B
**507.**	DHX29
**508.**	YME1L1
**509.**	RFC1
**510.**	ZYG11B
**511.**	RAD17
**512.**	KIF14
**513.**	ZNHIT6
**514.**	RASA1
**515.**	PSIP1
**516.**	LMBRD2
**517.**	INTS2
**518.**	TCERG1
**519.**	NSUN3
**520.**	KIF20B
**521.**	KIF21A
**522.**	HOOK1
**523.**	USP24
**524.**	ASPM
**525.**	ARIH1
**526.**	CCDC18
**527.**	YTHDC2
**528.**	PHAX
**529.**	ZNF227
**530.**	ZNF286A
**531.**	SENP1
**532.**	SCYL2
**533.**	PIK3CB
**534.**	CCDC15
**535.**	ZCCHC8
**536.**	CDC37L1
**537.**	TOPBP1
**538.**	DDHD1

**539.**	GEN1
**540.**	RBBP5
**541.**	UTP20
**542.**	CWF19L2
**543.**	LIN54
**544.**	QSER1
**545.**	MPHOSPH9
**546.**	ZNF283
**547.**	MBD4
**548.**	SGMS2
**549.**	SBNO1
**550.**	SEPSECS
**551.**	ZNF391
**552.**	SNAPC3
**553.**	FAF2
**554.**	PIGB
**555.**	LEO1
