# Supplementary material for: Identification of Gene Co-Expression Networks Associated with Consensus Molecular Subtype-1 of Colorectal Cancer
Source: Cancers (Basel). 2021 Nov 20;13(22):5824. doi: 10.3390/cancers13225824 (PMC8616344; doi:10.3390/cancers13225824)
Supplement: Supplementary file 1 [file cancers-13-05824-s001.zip › Table S2 Patient stages.pdf]

TCGA Patient IDs	TCGA Tumor Stage	Integromics Patient IDs	Integromics Tumor Stage
TCGA.AA.3562	3	IT001	3
TCGA.AA.3525	3	IT002	3
TCGA.AA.A00L	2	IT003	4
TCGA.AA.3672	3	IT004	3
TCGA.AA.3662	4	IT005	3
TCGA.G4.6321	3	IT006	3
TCGA.AA.3814	2	IT007	3
TCGA.AA.3554	2	IT008	3
TCGA.AA.3530	1	IT009	3
TCGA.AD.6888	3	IT010	3
TCGA.AA.3860	3	IT011	3
TCGA.AA.3506	1	IT012	4
TCGA.G4.6625	2	IT013	3
TCGA.AA.3552	3	IT014	3
TCGA.AA.3715	2	IT015	4
TCGA.G4.6310	3	IT016	3
TCGA.AA.3516	3	IT017	3
TCGA.AA.3697	2	IT018	3
TCGA.AA.3972	4	IT019	3
TCGA.AA.3556	1	IT020	3
TCGA.AA.3521	2	IT021	3
TCGA.G4.6314	4	IT022	4
TCGA.AZ.6601	2	IT023	3
TCGA.AA.3524	2	IT024	4
TCGA.AA.A024	2	IT025	4
TCGA.AA.3870	4	IT026	3
TCGA.AZ.6608	3	IT027	4
TCGA.AA.3543	1	IT028	3
TCGA.AA.3976	3	IT029	3
TCGA.CK.5912	1	IT030	3
TCGA.AA.3527	2	IT031	3
TCGA.AD.6964	0	IT032	2
TCGA.G4.6298	3	IT033	3
TCGA.G4.6586	2	IT034	3
TCGA.A6.4105	2	IT035	3
TCGA.AA.3675	2	IT036	3
TCGA.AA.3688	4	IT037	3
TCGA.AA.3950	2	IT038	3
TCGA.AA.3844	3	IT039	3
TCGA.AA.3867	4	IT040	4
TCGA.AA.A00Z	2	IT041	3
TCGA.AA.3692	4	IT042	3
TCGA.AA.3939	2	IT043	3
TCGA.AA.3522	2	IT044	3

TCGA.A6.6649	3	IT045	3
TCGA.AA.3979	2	IT046	3
TCGA.A6.2682	4	IT047	3
TCGA.AA.3952	3	IT048	3
TCGA.AA.3862	2	IT049	3
TCGA.A6.2677	3	IT050	3
TCGA.CM.5863	3	IT051	3
TCGA.G4.6588	2	IT052	3
TCGA.AA.3811	3	IT053	3
TCGA.AD.6895	3	IT054	2
TCGA.AA.3496	2	IT055	3
TCGA.AZ.4313	1	IT056	3
TCGA.AA.3713	4	IT057	3
TCGA.AY.4071	1	IT058	4
TCGA.AA.3531	2	IT059	3
TCGA.DM.A285	4	IT060	4
TCGA.AA.3986	1	IT061	3
TCGA.CM.4750	3	IT062	3
TCGA.AD.6548	1	IT063	4
TCGA.G4.6628	1	IT064	3
TCGA.G4.6294	4	IT065	3
TCGA.AA.3560	3	IT066	3
TCGA.G4.6317	3	IT067	3
TCGA.QG.A5YV	3	IT068	3
TCGA.AA.A01V	1	IT069	2
TCGA.A6.5665	2	IT070	3
TCGA.AA.3855	1	IT071	3
TCGA.AD.5900	1	IT072	4
TCGA.AA.3833	2	IT073	3
TCGA.AA.3511	2	IT074	3
TCGA.AA.3514	1	IT075	3
TCGA.AA.3956	2	IT076	4
TCGA.AA.A00Q	3	IT077	4
TCGA.AA.3875	1	IT078	3
TCGA.AA.A00N	2	IT079	3
TCGA.AA.3660	2	IT080	3
TCGA.CK.5916	1	IT081	3
TCGA.G4.6307	3	IT082	3
TCGA.CK.6746	2	IT083	3
TCGA.AA.3555	2	IT084	2
TCGA.QG.A5YX	2	IT085	3
TCGA.CK.6751	1	IT086	3
TCGA.D5.6531	2	IT087	3
TCGA.AA.3994	3	IT088	3
TCGA.AZ.6605	3	IT089	3

TCGA.AA.A01P	3	IT090	3
TCGA.CM.6675	4	IT091	3
TCGA.AA.A00D	1	IT092	3
TCGA.AA.3549	1	IT093	3
TCGA.CM.4751	3	IT094	3
TCGA.D5.5541	3	IT095	3
TCGA.DM.A1D6	2	IT096	3
TCGA.AZ.4308	3	IT097	3
TCGA.AY.A71X	1	IT098	3
TCGA.F4.6854	2	IT099	3
TCGA.G4.6299	3	IT100	3
TCGA.AU.6004	1	IT101	3
TCGA.D5.6533	0	IT102	3
TCGA.WS.AB45	2	IT103	4
TCGA.AA.3861	2	IT104	3
TCGA.4T.AA8H	2	IT105	2
TCGA.AA.3526	1	IT106	3
TCGA.A6.5656	1	IT107	3
TCGA.DM.A1D7	2	IT108	3
TCGA.CA.6718	2	IT109	3
TCGA.AA.A01K	3	IT110	4
TCGA.AA.3561	2	IT111	3
TCGA.AA.3877	1	IT112	3
TCGA.AZ.4614	4	IT113	3
TCGA.5M.AAT5	0	IT114	3
TCGA.AA.3941	4	IT115	3
TCGA.F4.6463	2	IT116	4
TCGA.CM.6170	1	IT117	3
TCGA.F4.6807	3	IT118	4
TCGA.AD.6965	3	IT119	4
TCGA.D5.6920	2	IT120	2
TCGA.AA.A01T	3	IT121	3
TCGA.F4.6460	3	IT122	3
TCGA.AZ.4615	3	IT123	4
TCGA.DM.A28M	2	IT124	3
TCGA.5M.AATE	2	IT125	3
TCGA.AU.3779	2	IT126	3
TCGA.CM.4746	1	IT127	3
TCGA.CM.5349	2	IT128	3
TCGA.AA.3989	4	IT129	3
TCGA.D5.6535	3	IT130	3
TCGA.AA.3502	1	IT131	3
TCGA.DM.A282	2	IT132	3
TCGA.DM.A1D0	2	IT133	2
TCGA.CA.6715	3	IT134	4

TCGA.AA.A022	2	IT135	3
TCGA.AD.A5EJ	2	IT136	3
TCGA.AA.3819	2	IT137	3
TCGA.A6.A565	3	IT138	3
TCGA.A6.6651	3	IT139	3
TCGA.G4.6320	3	IT140	3
TCGA.AA.3693	4	IT141	4
TCGA.CM.6162	3	IT142	3
TCGA.AY.A8YK	4	IT143	3
TCGA.AA.3553	1	IT144	4
TCGA.CM.6164	2	IT145	3
TCGA.AZ.5407	1	IT146	3
TCGA.DM.A28C	2	IT147	3
TCGA.A6.5667	3	IT148	3
TCGA.A6.2675	2	IT149	4
TCGA.AA.A01R	3	IT150	3
TCGA.AA.3679	4	IT151	3
TCGA.G4.6322	3	IT152	3
TCGA.D5.6928	2	IT153	3
TCGA.A6.2678	3	IT154	3
TCGA.DM.A1D9	2	IT155	2
TCGA.A6.6781	3	IT156	3
TCGA.AA.A017	2	IT157	3
TCGA.AA.3852	2	IT158	3
TCGA.5M.AATA	0	IT159	3
TCGA.A6.4107	3	IT160	3
TCGA.AA.A004	2	IT161	4a
TCGA.D5.6932	2	IT162	3
TCGA.A6.6780	2	IT163	3
TCGA.AA.3509	2	IT164	3
TCGA.AA.3821	1	IT165	3
TCGA.CA.5256	2	IT166	4a
TCGA.AA.A01X	3	IT167	3
TCGA.F4.6704	3	IT168	4a
TCGA.CM.6167	3	IT169	3
TCGA.QG.A5YW	3	IT170	3
TCGA.CA.5254	2	IT171	3
TCGA.D5.6927	2	IT172	3,1
TCGA.A6.5657	3	IT173	1
TCGA.SS.A7HO	0	IT174	3
TCGA.AY.5543	4	IT175	3
TCGA.AA.A03J	1	IT176	2
TCGA.AA.A00U	3	IT177	3
TCGA.D5.6898	1	IT178	3
TCGA.G4.6311	3	IT179	3

TCGA.F4.6806	1	IT180	3
TCGA.AA.A01Z	2	IT181	4
TCGA.G4.6306	0	IT182	3
TCGA.AA.3678	3	IT183	3
TCGA.CA.6719	3	IT184	3
TCGA.CM.5341	2	IT185	3
TCGA.AA.3548	3	IT186	3
TCGA.AA.3681	3	IT187	3
TCGA.AA.A01Q	2	IT188	3
TCGA.NH.A8F7	2	IT189	3
TCGA.AZ.6607	4	IT190	3
TCGA.F4.6703	2	IT191	3
TCGA.AA.3685	2	IT192	3
TCGA.DM.A1HA	3	IT193	3
TCGA.A6.2683	4	IT194	3
TCGA.D5.6534	2	IT195	3
TCGA.AZ.4684	4	IT196	4
TCGA.A6.6141	2	IT197	3
TCGA.D5.6929	4	IT198	1
TCGA.A6.2680	2	IT199	4
TCGA.AA.A00K	2	IT200	3
TCGA.NH.A6GA	3	IT201	3
TCGA.D5.6538	3	IT202	2
TCGA.AA.3968	1	IT203	4a
TCGA.AA.A02W	1	IT204	3
TCGA.CM.6676	1	IT205	3
TCGA.AA.A01F	3	IT206	3
TCGA.D5.6922	3	IT207	3
TCGA.AA.A02K	4	IT208	3
TCGA.AA.A00J	3	IT209	3
TCGA.AA.3854	1	IT210	3
TCGA.A6.2681	2	IT211	3
TCGA.CM.4744	1	IT212	4b
TCGA.CM.5860	2	IT225	4
TCGA.CM.4747	4	IT230	3
TCGA.NH.A50U	4	IT226	3
TCGA.AA.3510	2	IT215	3
TCGA.AA.3494	4	IT231	3
TCGA.AY.A69D	2	IT216	4
TCGA.CK.4951	2	IT217	4
TCGA.DM.A288	3	IT227	3
TCGA.CA.5255	2	IT232	3
TCGA.CM.6168	2	IT218	4
TCGA.D5.6536	2	IT213	4
TCGA.NH.A6GB	3	IT219	3

TCGA.AA.3684	4	IT236	4
TCGA.CM.6161	1	IT228	4
TCGA.AZ.6603	0	IT220	4
TCGA.AD.6963	0	IT229	4
TCGA.AA.A010	0	IT233	4
TCGA.A6.5659	1	IT221	3
TCGA.F4.6855	2	IT234	3
TCGA.D5.5540	2	IT222	3
TCGA.G4.6297	4	IT223	3
TCGA.CM.4743	2	IT214	4
TCGA.CM.4752	2	IT224	4
TCGA.AA.A00W	1	IT235	4b
TCGA.AA.3980	1	IT237	4, 1
TCGA.DM.A28K	2	IT238	2
TCGA.AM.5821	2	IT239	3
TCGA.AA.3971	3	IT240	3
TCGA.NH.A5IV	2	IT241	4
TCGA.AA.3831	2	IT242	3
TCGA.QG.A5Z2	1	IT243	4b
TCGA.AZ.4616	4	IT244	4
TCGA.DM.A280	2	IT245	3
TCGA.AA.3812	2	IT246	2
TCGA.A6.2685	2	IT247	2
TCGA.AA.3973	4	IT248	3
TCGA.CM.5862	4	IT249	3
TCGA.DM.A0X9	2	IT250	3
TCGA.DM.A1HB	3	IT251	3
TCGA.CA.6716	2	IT252	3
TCGA.AA.A02O	2	IT253	3
TCGA.AA.3966	2	IT254	3
TCGA.AA.3673	2	IT255	3
TCGA.AA.3848	3	IT256	3
TCGA.D5.6923	1	IT257	4b
TCGA.A6.5666	2	IT258	3
TCGA.D5.5538	3	IT259	3
TCGA.DM.A1D8	0	IT260	3
TCGA.F4.6808	1	IT261	3
TCGA.F4.6461	3		
TCGA.AA.A00O	3		
TCGA.CM.6169	2		
TCGA.AA.3866	1		
TCGA.CM.6678	4		
TCGA.AA.3837	2		
TCGA.AY.4070	3		
TCGA.D5.6530	1		

TCGA.D5.5537	0
TCGA.AD.6889	2
TCGA.CK.4947	3
TCGA.AA.A00E	2
TCGA.F4.6569	1
TCGA.QG.A5Z1	3
TCGA.AZ.6598	2
TCGA.F4.6570	2
TCGA.AA.3955	3
TCGA.DM.A1DB	2
TCGA.CM.4748	3
TCGA.CM.5348	3
TCGA.A6.5661	2
TCGA.AA.A00F	3
TCGA.AD.A5EK	1
TCGA.G4.6323	0
TCGA.D5.6529	2
TCGA.AA.3970	2
TCGA.A6.2686	2
TCGA.CM.6171	1
TCGA.AA.3538	1
TCGA.D5.6539	0
TCGA.AA.3984	2
TCGA.D5.6540	1
TCGA.DM.A28H	3
TCGA.AA.3818	2
TCGA.D5.6931	3
TCGA.AA.3930	4
TCGA.G4.6304	2
TCGA.AA.A03F	3
TCGA.AA.A00R	1
TCGA.G4.6295	2
TCGA.AD.6890	0
TCGA.CM.6163	1
TCGA.AA.3666	3
TCGA.A6.6142	4
TCGA.AA.3488	4
TCGA.AA.3667	1
TCGA.A6.6650	2
TCGA.CM.6165	2
TCGA.AZ.4682	4
TCGA.A6.3810	2
TCGA.CM.6166	1
TCGA.A6.A566	3
TCGA.DM.A1D4	2

TCGA.AA.3663	2
TCGA.D5.6537	3
TCGA.CM.6679	2
TCGA.5M.AAT4	4
TCGA.A6.2684	1
TCGA.DM.A28G	2
TCGA.A6.6140	2
TCGA.D5.6532	2
TCGA.DM.A28E	2
TCGA.AA.3655	2
TCGA.AA.A01G	2
TCGA.AA.3975	1
TCGA.G4.6293	3
TCGA.NH.A50V	3
TCGA.A6.2676	2
TCGA.NH.A8F8	4
TCGA.AZ.4315	2
TCGA.G4.6315	4
TCGA.A6.6652	4
TCGA.AA.3712	3
TCGA.AA.3842	3
TCGA.A6.6138	1
TCGA.A6.3807	3
TCGA.AA.3949	3
TCGA.D5.6930	2
TCGA.AA.3869	4
TCGA.CM.5344	3
TCGA.D5.6924	2
TCGA.AD.6901	0
TCGA.DM.A0XD	2
TCGA.D5.7000	1
TCGA.5M.AAT6	4
TCGA.CK.6748	4
TCGA.D5.5539	3
TCGA.CK.5913	2
TCGA.AA.3846	2
TCGA.RU.A8FL	3
TCGA.AA.A02E	4
TCGA.A6.2672	3
TCGA.CA.6717	2
TCGA.AA.A01S	3
TCGA.AA.3864	2
TCGA.AY.6197	2
TCGA.AA.3815	2
TCGA.AZ.4323	4

TCGA.AA.3841	2
TCGA.AA.3858	0
TCGA.CK.4948	3
TCGA.AA.3680	4
TCGA.CK.4950	3
TCGA.NH.A50T	2
TCGA.A6.2671	4
TCGA.AZ.6600	4
TCGA.CK.4952	3
TCGA.G4.6302	2
TCGA.AY.A54L	1
TCGA.A6.3809	2
TCGA.AD.6899	3
TCGA.AA.3851	2
TCGA.AA.A01D	3
TCGA.CA.5797	2
TCGA.AA.A02Y	1
TCGA.CK.5915	1
TCGA.CM.5868	4
TCGA.A6.2679	2
TCGA.A6.6654	3
TCGA.AA.3856	2
TCGA.AA.3534	2
TCGA.AA.3529	3
TCGA.AZ.5403	2
TCGA.AA.3520	2
TCGA.AA.A02R	2
TCGA.4N.A93T	3
TCGA.AA.3872	4
TCGA.F4.6805	2
TCGA.AA.3947	2
TCGA.AA.A02H	4
TCGA.DM.A1DA	3
TCGA.A6.6648	4
TCGA.A6.3808	2
TCGA.AA.3977	0
TCGA.AA.A01C	3
TCGA.AA.3518	2
TCGA.CM.6674	2
TCGA.AA.3489	2
TCGA.AA.3519	3
TCGA.AA.3664	2
TCGA.AA.3492	2
TCGA.AA.A029	2
TCGA.CM.6172	3

TCGA.AA.3696	4
TCGA.DM.A28F	3
TCGA.3L.AA1B	1
TCGA.AA.A00A	2
TCGA.AZ.6606	4
TCGA.A6.6137	3
TCGA.AA.3982	3
TCGA.AA.A02J	4
TCGA.A6.A567	4
TCGA.F4.6809	4
TCGA.AA.3710	2
TCGA.CK.6747	2
TCGA.F4.6459	3
TCGA.CM.5864	1
TCGA.AA.3542	3
TCGA.A6.6653	1
TCGA.D5.6926	3
TCGA.CK.5914	3
TCGA.A6.A5ZU	3
TCGA.A6.5660	3
TCGA.A6.5662	4
TCGA.G4.6303	4
TCGA.DM.A0XF	3
TCGA.G4.6309	3
TCGA.F4.6856	1
TCGA.AA.A01I	1
TCGA.G4.6627	2
TCGA.CM.6677	2
TCGA.AY.6196	3
TCGA.AA.3532	2
TCGA.A6.2674	4
TCGA.QL.A97D	1
TCGA.AA.3517	2
TCGA.AA.3845	2
TCGA.CM.6680	3
TCGA.AM.5820	4
TCGA.DM.A28A	3
TCGA.AA.3495	1
TCGA.D5.6541	2
TCGA.AA.3850	1
TCGA.NH.A6GC	4
TCGA.AZ.6599	0
TCGA.G4.6626	2
TCGA.CA.5796	2
TCGA.CM.5861	2

TCGA.A6.A56B	3
TCGA.AA.3544	1
TCGA.A6.5664	3
TCGA.AA.A02F	4
TCGA.AY.6386	3
TCGA.T9.A92H	2
TCGA.A6.6782	2
